# An Aqueous Extract of *Tuberaria lignosa* Inhibits Cell Growth, Alters the Cell Cycle Profile, and Induces Apoptosis of NCI-H460 Tumor Cells

**DOI:** 10.3390/molecules21050595

**Published:** 2016-05-06

**Authors:** Joana M. Pereira, Vanessa Lopes-Rodrigues, Cristina P. R. Xavier, M. João Lima, Raquel T. Lima, Isabel C. F. R. Ferreira, M. Helena Vasconcelos

**Affiliations:** 1 Department of Biological Sciences, FFUP—Faculty of Pharmacy of the University of Porto, Rua de Jorge Viterbo Ferreira 228, Porto 4050-313, Portugal; joanita_12@live.com.pt (J.M.P.); mjpclima@gmail.com (M.J.L.); 2 i3S—Instituto de Investigação e Inovação em Saúde da Universidade do Porto, Rua Alfredo Allen 208, Porto 4200-135, Portugal; vrodrigues@ipatimup.pt (V.L.-R.); cristinax@ipatimup.pt (C.P.R.X.); rlima@ipatimup.pt (R.T.L.); 3 Cancer Drug Resistance Group, IPATIMUP—Institute of Molecular Pathology and Immunology of the University of Porto, Porto 4200-135, Portugal; 4 ICBAS-UP—Institute of Biomedical Sciences Abel Salazar, University of Porto, Porto 4050-313, Portugal; 5 Department of Pathology and Oncology, FMUP—Faculty of Medicine of the University of Porto, Alameda Prof. Hernâni Monteiro, Porto 4200-319, Portugal; 6 Mountain Research Centre (CIMO), Polytechnic Institute of Bragança, Apartado 1172, Bragança 5301-855, Portugal

**Keywords:** *T. lignosa* extracts, inhibition of tumor cell growth, H460 tumor cell line, cell cycle, apoptosis

## Abstract

*Tuberaria lignosa* (Sweet) Samp. is found in European regions, and has antioxidant properties due to its composition in ascorbic acid and phenolic compounds. Given its traditional use and antioxidant properties, the tumor cell growth inhibitory potential of aqueous extracts from *T. lignosa* (prepared by infusion and decoction) was investigated in three human tumor cell lines: MCF-7 (breast adenocarcinoma), NCI-H460 (non-small cell lung cancer), and HCT-15 (human colorectal adenocarcinoma). Both extracts inhibited the growth of these cell lines; the most potent one being the *T. lignosa* extract obtained by infusion in the NCI-H460 cells (GI_50_ of approximately 50 μg/mL). Further assays were carried out with this extract in NCI-H460 cells. At 100 μg/mL or 150 μg/mL it caused an increase in the percentage of cells in the G0/G1 phase and a decrease of cells in S phase of the cell cycle. Additionally, these concentrations caused an increase in the percentage of apoptotic cells. In agreement, a decrease in total poly (ADP-ribose) polymerase (PARP) and pro-caspase 3 levels was found. In conclusion, the *T. lignosa* extract obtained by infusion was more potent in NCI-H460 cells, altering the cell cycle progression and inducing apoptosis. This work highlights the importance of *T. lignosa* as a source of bioactive compounds with tumor cell growth inhibitory potential.

## 1. Introduction

Herbal drugs have been extensively used for the treatment of several diseases all over the world [1,2,3]. In particular, the interest in plants as a natural source of pharmaceutical compounds with antitumor activity has increased in the past years. Indeed, several anti-cancer agents derived from plants (such as vinca alkaloids, epipodophyllotoxins derivatives, taxanes, and the campothecin derivatives) have been discovered in the last decades [1,2,4,5,6,7,8,9]. Furthermore, several plant-derived anticancer agents have achieved pre-clinical or clinical development [7,8,10,11].

*Tuberaria lignosa* (Sweet) Samp. (Rockrose like species) is a plant found in the west and south part of Europe and is mostly present in the Iberian Peninsula, where it is known as “alcária” or “erva loba” [12,13]. This plant has been used in traditional medicine to treat several diseases, such as gastrointestinal disorders, viral infections, and skin infections, among others [12,13]. The preparation of medicinal infusions and decoctions may involve the use of the whole plant or just some parts of the plant and may be prepared fresh or shade-dried [12]. The chemical composition of *T. lignosa* aqueous extracts, obtained by infusion and decoction, has been previously analyzed and compared by some of the authors [12,13]. It has been demonstrated that infusions obtained from freeze-dried samples have higher concentrations in compounds such as free sugars (e.g., fructose, glucose, sucrose, trehalose, and raffinose) and lower concentrations of ascorbic acid and phenolic compounds (including ellagic acid derivatives and flavonoids) when compared with decoctions obtained from freeze-dried samples and infusions prepared from shade-dried samples [12]. Additionally, the freeze-dried samples also proved to have higher antioxidant activity than the other samples and even higher than trolox, the positive control used in the referred study [12]. Both infusion and decoction samples from *T. lignosa* have bioactive molecules, such as phenolic compounds and ascorbic acid, which can explain their antioxidant activity [12,13,14,15].

In spite of its content in such bioactive molecules, to our knowledge the tumor cell growth inhibitory potential of this plant has never been studied. Therefore, the main objective of this work was to investigate the tumor cell growth inhibitory effect of two aqueous extracts prepared by infusion and decoction from *Tuberaria lignosa* in different human tumor cell lines: MCF-7 (breast adenocarcinoma), NCI-H460 (non-small cell lung cancer), and HCT-15 (human colorectal adenocarcinoma). In addition, the cellular effects of the *T. lignosa* extract obtained by infusion on cell cycle profile and apoptosis were studied in more detail in the most sensitive cell line (NCI-H460).

## 2. Results and Discussion

### 2.1. T. lignosa Extracts Inhibited Tumor Cell Growth

The sulforhodamine B (SRB) assay was carried out in order to determinate the *in vitro* cytotoxicity of the *T. lignosa* aqueous extracts, prepared by decoction and infusion, in three different human tumor cell lines, using doxorubicin as the positive control. The results showed that *T. lignosa* extract obtained by infusion was the most potent extract in the MCF-7 and in the NCI-H460 cells (Table 1). However, in the HCT-15 cells both extracts had similar effect. Overall, the most potent extract was the infusion of *T. lignosa* in the NCI-H460 cells. This is probably due to the different chemical composition of the extracts, namely in phenolic acid derivatives, ellagic acid derivatives, and flavonoids, with infusion presenting higher content in phenolic acid derivatives [12]. Therefore, the following analyses were conducted with this extract (infusion). The cell line selected to continue the studies was the NCI-H460, since this was the cell line in which the infusion extract was more potent and since it is representative of lung cancer which has high incidence and is still one of the most common cause of cancer related deaths [16].

The compounds isolated from plants with potential activity against cancer have shown multiple mechanisms of action, such as interference with death signals thereby inducing apoptosis, disturbance of multiple cellular signaling pathways, regulation of the cell cycle, and/or interference with cellular invasion and metastasis [3,17,18,19,20,21]. For this reason, this study has further investigated the effect of the infusion extract of *T. lignosa* on cell cycle and apoptosis.

### 2.2. T. lignosa Infusion Extract Blocked the Cell Cycle Progression of NCI-H460 Cells

**Figure 1:**
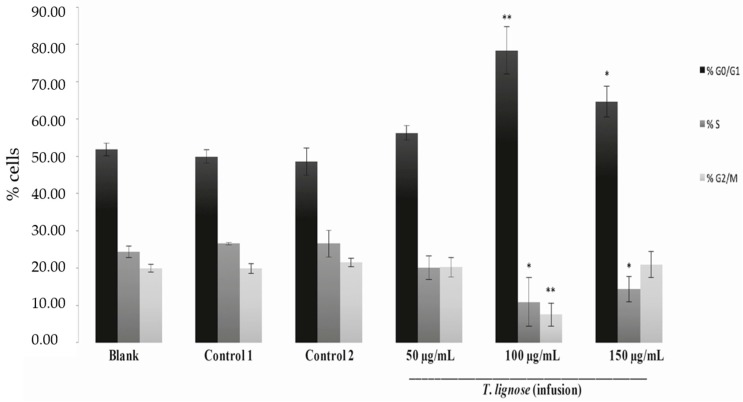
Effect of *T. lignosa* infusion extract on NCI-H460 cell cycle distribution. Cells were treated for 48 h with complete medium (Blank), with the extract (50 µg/mL, 100 µg/mL, or 150 µg/mL), or with Control treatments (the two highest vehicle, H_2_O, concentrations). Results are the mean ± SEM of four independent experiments. * *p* ≤ 0.05 and ** *p* ≤ 0.01 Blank *vs.* Treatment.

To gain insight into the cytotoxic mechanism of action of the *T. lignosa* extract obtained by infusion, its effect on the cell cycle was analyzed. For this, to assess a possible dose-dependent effect, NCI-H460 cells were treated with increasing concentrations of the extract: 50 μg/mL, 100 μg/mL, and 150 μg/mL (approximately the GI_50_, 2 × GI_50,_ and 3 × GI_50_ concentrations, respectively, with GI referring to the concentration that inhibits 50% of net cell growth). Controls for the solvent of the extract (H_2_O) were included. Results showed that treatment of the NCI-H460 cells with the highest tested concentrations (100 μg/mL or 150 μg/mL) of the extract caused a statistically significant increase in the percentage of cells in the G0/G1 phase of the cell cycle and a statistically significant decrease in the percentage of the cells in the S phase of the cell cycle (Figure 1). In addition, for the 100 μg/mL concentration a statistically significant decrease in the G2/M phase of the cell cycle was also observed. This data suggested that *T. lignosa* infusion extract blocked the cell cycle progression of the NCI-H460 cells, having a significant effect at 100 μg/mL and 150 μg/mL.

Various plant extracts with recognized antitumor activity have similar effects, arresting cells in the G0/G1 phase of the cell cycle, such as ginseng extract [22], the extract of *Tinospora cordifolia* [23], or the *Marsdenia tenacissima* extract [24].

### 2.3. T. lignosa Infusion Extract Induced Apoptosis in NCI-H460 Cells

The effect of *T. lignosa* infusion extract on cellular apoptosis was also analyzed by flow cytometry analysis of Annexin V-FITC and propidium iodide (PI) labeling/staining. Results (Table 2) showed an increase in the percentage of cells undergoing apoptosis after treatment with the extract, with statistically significant results observed for the 150 μg/mL concentration treatment. Treatment of cells with 100 μg/mL of extract also caused an increase in apoptosis to nearly twice the control levels, even though this increase was not considered statistically significant. The reason why cellular treatment with 100 μg/mL of extract caused statistically significant alterations in cell cycle but not in apoptosis is possibly because the effect on the cell cycle might precede the effect on apoptosis. Time-course experiments would confirm this hypothesis. Nevertheless, overall this data demonstrated that this extract caused apoptosis in NCI-H460 cells.

**Figure 2:**
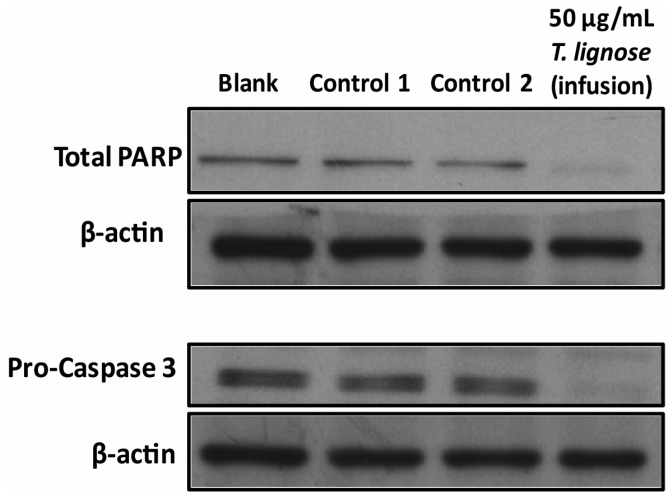
Effect of *T. lignosa* infusion extract on the NCI-H460 cellular expression levels of total PARP-1 and pro-caspase-3, analyzed by Western blot. Cells were treated for 48 h with complete medium (Blank), Controls (vehicle of the extract, H_2_O), or with 50 µg/mL of the extract. β-actin was used as loading control. Blots are representative of two independent experiments.

To confirm the effect on apoptosis, the effect of this extract on the expression levels of cellular proteins involved in apoptosis was analyzed. Results from the Western blot analysis showed a decrease in the levels of total poly (ADP-ribose) polymerase (PARP) and pro-caspase 3 (Figure 2). These results further confirm that the *T. lignosa* infusion extract induced apoptosis in NCI-H460 cells. Indeed, a reduction in the total levels of PARP is an accepted event in cellular apoptosis [25,26]. Unfortunately, our antibody does not detect the cleaved form of caspase-3. However, the observed reduction in pro-caspase 3 is indicative that the extract induced the intrinsic pathway of apoptosis [27,28].

## 3. Material and Methods

### 3.1. Samples and Preparation of the T. lignosa Extracts

*T. lignosa* was collected in Miranda do Douro (Trás-os-Montes, Northeastern Portugal), as referred in Pinela *et al.,* 2012 [12]. The preparation of decoctions and infusions followed the protocol previously described by Pinela *et al.*, 2012 [12]. Stock solutions of the extract were prepared in water and stored at −20 °C. Both aqueous extracts were previously characterized by some of the authors in terms of phenolic compounds [12].

### 3.2. Cell Culture

Three different human tumor cell lines were used: MCF-7 (breast adenocarcinoma), NCI-H460 (non-small cell lung cancer), and HCT-15 (human colorectal adenocarcinoma). All of these cells were maintained in RPMI-1640 medium with Glutamax™, supplemented with 1 M 4-(2-Hydroxyethyl)piperazine-1-ethanesulfonic acid (HEPES) buffer and fetal bovine serum (FBS). The FBS concentration used was 5% for the cell growth inhibition assay and 10% for the remaining assays. The monolayer cultures were kept at 37 °C in a humidified incubator with 5% CO_2_. Cells were routinely observed with a microscope (Nikon eclipse TS100 microscope, Nikon Instruments Europe BV, Amsterdam, The Netherlands). All the experiments described were performed only when exponentially growing cells presented more than 90% viability.

### 3.3. Cell Growth Inhibition Assay

The SRB assay was adapted from the procedure used in the NCI’s *in vitro* anti-cancer drug screening [29,30]. Briefly, cells were plated in 96-well plates at their previously determined optimal concentrations (5.0 × 10^4^ cells/mL for NCI-H460 and MCF-7 cells and 1.0 × 10^5^ cells/mL for HCT-15 cells) and incubated for 24 h. Cells were then treated with five serial dilutions of *T. lignosa* infusion and *T. lignosa* decoction, ranging from 400 µg/mL to 25 µg/mL. Doxorubicin was used as a positive control (ranging from 150 nM to 9.37 nM). The effect of the solvent of the extracts (water) on the growth of the cell lines was also evaluated by treating cells with the maximum concentration of water used. Following 48 h incubation with the extract, plates were fixed by adding ice-cold 10% ice cold trichloroacetic acid (TCA) (*w*/*v*, final concentration, Panreac, Barcelona, Spain) and stained with 1% SRB (Sigma Aldrich, St. Louis, MO, USA) in 1% (*v*/*v*) acetic acid. Bound dye was solubilized by adding 10 mM Tris base solution (Sigma Aldrich) and finally the absorbance was measured at 510 nm in a microplate reader (BioTek^®^ Synergy HT, Winooski, VT, USA). Using the SRB assay, the GI_50_ concentration (concentration that inhibits 50% of net cell growth) was determined for *T. lignosa* infusion and decoction extracts in each cell line.

### 3.4. Preparation of Cells for Other Analyses

For the analysis of the cell cycle, apoptosis, or Western blot, the NCI-H460 cells were plated at 1.5 ×10^5^ cells/well in-well plates and incubated for 24 h. Cells were then treated with different concentrations of the *T. lignosa* infusion extract: 50 μg/mL (approximately the GI_50_ concentration), 100 μg/mL (approximately twice the GI_50_), and/or 150 μg/mL (approximately three times the GI_50_). Blank cells (treated with medium) and Control cells (treated with a concentration of water corresponding to the treatment with 100 μg/mL—Control 1, or 150 μg/mL—Control 2) were also included. After treatment, cells were trypsinized and centrifuged at 1200 rpm for 5 min. Cells were then processed according to the procedures described below.

### 3.5. Analysis of Cell Cycle Profile

Cells were washed in PBS, fixed with ice-cold 70% ethanol, and stored at 4 °C for at least 12 h. Next, the cells were centrifuged (5 min at 1200 rpm) and the pellets were re-suspended in a solution of PBS containing 5 μg/mL propidium iodide and 0.1 mg/mL RNase A. Cellular DNA content was analyzed by flow cytometry and the percentage of cells in the G0/G1, S, and G2/M phases of the cell cycle were determined using the BD Accuri™ C6 Flow cytometer (BD Biosciences) after cell debris and aggregates exclusion and plotting at least 20,000 events per sample [31,32]. All data was analyzed using the FlowJo software (version 7.6.5, Tree Star, Inc., Ashland, OR, USA).

### 3.6. Analysis of Apoptosis

For the analysis of apoptosis, cell pellets were washed and re-suspended in 1 mL of PBS. Following centrifugation, cells were processed for apoptosis analysis with the Annexin V-FITC Apoptosis Detection Kit (eBioscience) by resuspension in buffer solution, as indicated by the manufacturer. Cells were then incubated for 10 min with Human Annexin V-FICT and further incubated for 5 min with propidium iodide. Cells were analyzed by flow cytometry using the BD Accuri™ C6 Flow cytometer and respective software, plotting at least 20,000 events per sample [33,34]

### 3.7. Analysis of Protein Expression

For the analysis of protein expression, cell pellets were washed and resuspended in PBS. Cells were centrifuged and pellets were stored at −20 °C. Cell pellets were then lysed in Winman’s buffer containing 1% NP-40, 0.1 M Tris–HCl pH 8.0, 0.15 M NaCl, and 5 mM EDTA, complemented with protease inhibitor cocktail (Roche). The total protein content was quantified using the DC™ Protein Assay kit (Bio-Rad, Hercules, CA, USA) according to manufacturer’s instructions. Protein lysate (20 µg) were loaded on 12% SDS-PAGE gel and transferred into a nitrocellulose membrane (GE Healthcare, Cleveland, OH, USA). The following primary antibodies were used: rabbit anti-PARP-1 (1:2000, sc-7150, Santa Cruz Biotechnology, Heidelberg, Germany), mouse anti-Caspase 3 (1:2000, 05-654, Merck Millipore, Darmstadt, Germany), and goat anti-actin (1:2000, sc-1616, Santa Cruz Biotechnology). The corresponding secondary antibodies were: anti-rabbit IgG-HRP, anti-mouse IgG-HRP, or anti-goat IgG-HRP (1:2000, Santa Cruz Biotechnology). The Amersham™ ECL Western Blotting Detection Reagents (GE Healthcare), the High Performance Chemiluminescence Film (GE Healthcare), and the Kodak GBX developer and fixer (Sigma) were used for signal detection [35,36].

### 3.8. Statistical Analysis

Three replicates of each aqueous extract were used. The data was statistically analyzed using the two-tailed paired Student’s *t*-test.

## 4. Conclusions

The obtained results show that both infusion and decoction extracts from the plant *T. lignosa* caused reduction in the growth of three human tumor cell lines. To our knowledge, this is the first report of such an activity for this plant. The most potent effect was observed with the infusion extract in the NCI-H460 cells. The effect of this extract on these cells was due to alterations in the cell cycle profile and to induction of cellular apoptosis. Therefore, the data here presented highlights the interest of this plant as a possible source of natural bioactive compounds with tumor cell growth inhibitory potential. It would be interesting to isolate the compounds present in the infusion extract of this plant and to identify which one(s) are responsible for the observed effects in this tumor cell line. It would also be of interest to extend this study to other extracts of this plant and other cell lines.

## Figures and Tables

**Table 1:** GI_50_ concentrations of *T. lignosa* extracts in three human tumor cell lines.

Extracts	GI_50_ Concentrations (µg/mL) in Different Cell Lines		
	MCF-7	HCT-15	NCI-H460
Decoction	135.1 ± 14.6	47.0 ± 2.2	103.7 ± 19.0
Infusion	72.9 ± 10.2	57.5 ± 8.2	43.4 ± 7.8

GI_50_ concentrations correspond to the mean ± S.E. of at least three independent experiments, carried out with duplicates. Doxorubicin was used as a positive control, with the following results having been obtained: 56.3 nM ± 14.5 in MCF-7 cells and 89.3 nM ± 16.7 in NCI-H460 cells. GI refers to the concentration that inhibits 50% of net cell growth. S.E. refers to standard error.

**Table 2:** Apoptosis levels in NCI-H460 cells treated with infusion extract of *T. lignosa*.

Conditions		% Apoptosis
Blank		9.51 ± 0.9
Control (H_2_O)	Control 1	9.45 ± 0.3
	Control 2	12.7 ± 1.9
*T. lignosa* (infusion)	50 µg/mL	15.8 ± 7.1
	100 µg/mL	22.0 ± 6.1
	150 µg/mL	34.7 ± 7.9 *

Results are the mean ± SEM of three independent experiments. * *p* ≤ 0.05 blank *vs.* treatment. Control treatments correspond to the two highest vehicle (H_2_O) concentrations.
